# The Psychometric Properties for the VISIONS QL Brief

**DOI:** 10.3390/healthcare13233046

**Published:** 2025-11-25

**Authors:** Ali Brian, Pamela Beach, Andrea Taliaferro

**Affiliations:** 1 Department of Educational and Developmental Science, College of Education, University of South Carolina, Columbia, SC 29208, USA; taliafa@mailbox.sc.edu; 2 College of Health Sciences and Technology, Rochester Institute of Technology, Rochester, NY 14623, USA; psbchst@rit.edu

**Keywords:** visual impairment, quality of life, youth, validity, reliability

## Abstract

**Background/Objectives:** Children with visual impairments (VI) experience lower Quality of Life (QoL), higher sedentary time, and reduced motor competence as compared to their sighted peers, posing challenges to their health, well-being, and psychosocial development. While several QoL instruments have been developed internationally for children/youth with VI, none have been validated for use with U.S. pediatric populations. The purpose of this study was to evaluate the psychometric properties of the VISIONS QL assessment tool tailored for children/youth with VI, with a primary aim of variable/item reduction to develop a streamlined version of the instrument. **Methods:** This study featured a cross-sectional, descriptive analytic design with convenience sampling. Participants were children and youth with VI, aged 9–19 years, (*N* = 148; Boys = 71, Girls = 77; *M_age_* = 14.49, *SD* = 3.36 years). A principal components analysis (PCA) with orthogonal varimax rotation was conducted to reduce dimensionality and identify components. **Results:** Results of the PCA yielded three components explaining 46% of the variance: Educational Opportunities = 7 items; Social and Familial Implications = 8 items; Communication = 5 items. Overall, the VISIONS QL Brief had a high level of internal consistency reliability (α = 0.857; Ω = 0.858) and test–retest reliability (ICC = 0.89, 95% CI = 0.84–0.92). The original 63-item version showed concurrent validity with the 20-item brief scale (*r* = 0.92, *p* < 0.0001). **Conclusions:** Findings affirm the multidimensional nature of QoL and support the usage of the VISIONS QL Brief and its utility in settings where time, accessibility, and cognitive load are critical considerations.

## 1. Introduction

Childhood vision impairment, affecting approximately 6.8% of U.S. children under 18 with 3% categorized as blind or low vision [1], represents a critical public health concern due to its potential to disrupt developmental trajectories and diminish overall quality of life [2]. Quality of Life (QoL) in children encompasses physical, psychological, social, and school-related dimensions [3,4]. These domains reflect both subjective perceptions and objective indicators of well-being, including physical health (e.g., energy, mobility, and pain levels), emotional functioning (e.g., mood, anxiety, and self-esteem), social relationships (e.g., peer and family interactions), and school functioning (e.g., engagement, cognitive development, and academic performance). Children with visual impairments (VI) experience lower QoL compared to their sighted peers, particularly in psychological well-being and social relationships [5,6,7] (e.g., elevated rates of anxiety, lower self-confidence, and limited peer interaction [8,9]). Furthermore, children with VI typically experience higher sedentary time and reduced motor competence [10], posing additional challenges to their health and psychosocial development. Thus, assessing QoL in children is vital to understanding how health conditions influence not just clinical outcomes, but broader life satisfaction and developmental trajectories [11].

While several QoL instruments have been developed internationally for children/youth with VI, including the Vision-Related QoL Questionnaires for Children and Young People [12,13] and the Impact of Vision Impairment for Children (IVI_C) [14], none have been psychometrically vetted for use with U.S. pediatric populations. Most QoL instruments are designed for clinical settings and lack adaptation for broader educational or psychosocial contexts. For example, the Pediatric Quality of Life Inventory (PedsQL™) is a widely used, multidimensional tool assessing physical, emotional, social, and school functioning across diverse pediatric populations [15]. Though robust and translated into over 60 languages and extensively vetted in dozens of pediatric populations, it is a broad-based indicator and may not fully capture the nuanced experiences of youth with sensory impairments.

To address limitations in generic QoL measures for sensory impairments, Umansky et al. (2011) developed the HEAR-QL for children with hearing loss, showing greater sensitivity than the PedsQL™ in distinguishing between youth who are and are not impaired [16]. Building on its strong psychometric properties (e.g., Cronbach’s α > 0.88; *r* = 0.83), Beach et al. (2024) adapted the HEAR-QL into the VISIONS QL to assess the QoL among children with VI [17]. An expert panel conducted a two-round Delphi process to evaluate face and content validity of the VISIONS QL and adapted PedsQL™ items, using a four-point Likert scale. Items below a 3.25 median or with <70% agreement were revised. Most VISIONS QL items reached consensus in round one (*n* = 28); six required a second round. Despite promising development, further psychometric vetting is needed to ensure cultural relevance in U.S. populations.

Lengthy assessments can pose significant challenges for children/youth with disabilities due to increased cognitive load, accessibility barriers, and time constraints, often resulting in fatigue and compromised data quality [18]. In school, clinical, and research settings, shortened assessments offer practical advantages; they are quicker to administer, reduce burden on children/youth, and improve response rates, making them ideal for repeated use. To maintain the integrity of these tools, brief versions must undergo rigorous item selection and validation processes that preserve their psychometric strength, such as reliability and construct validity [19]. This approach ensures that the shortened versions continue to yield meaningful and accurate measures of QoL without compromising their original purpose.

Psychometric evaluation is essential for developing meaningful and accurate assessment tools, encompassing three core elements: reliability, validity, and factor structure [20]. Reliability ensures consistent outcomes across time and contexts, whether via internal consistency (e.g., Cronbach’s alpha) [21] or test–retest reliability. Validity determines whether a tool measures its intended construct, including content, construct, and criterion validity. Without validity, even consistent instruments may yield misleading conclusions. Factor structure reveals how items cluster to develop underlying dimensions, typically examined through exploratory and confirmatory factor analysis [22]. Together, these elements establish the scientific rigor and practical utility of psychometric instruments across diverse populations.

The purpose of this study was to evaluate the psychometric properties of the VISIONS QL assessment tool tailored for children and youth with VI to develop a streamlined version of the instrument. Refining VISIONS QL for clarity and relevance enhances its value as a reliable, evidence-based tool for clinicians, educators, and researchers. A psychometrically vetted, concise QoL measure supports early intervention by identifying psychosocial and functional challenges, guiding tailored support plans, and tracking outcomes over time. Its adaptability and ecological validity also make it useful in public health, education, and policy, promoting equitable access and informed decision-making for children and youth with VI.

## 2. Materials and Methods

### 2.1. Design, Participants, and Setting

This study featured a cross-sectional, descriptive analytic design with convenience sampling. Participants, aged 9–19 years (*N* = 148; Boys = 71, Girls = 77; *M_age_* = 14.49, *SD* = 3.36 years) included children and youth with visual impairments (B1 = 27%, B2 = 6%, B3 = 40%, B4 = 27%). Participants were classified according to the United States Association of Blind Athletes (USABA) visual impairment categories (B1–B4), which are determined by visual acuity and/or visual field in the better-seeing eye with best correction. The B1 category includes individuals with no light perception in either eye or some light perception but who are unable to recognize the shape of a hand at any distance or in any direction. B2 athletes can recognize the shape of a hand and/or have a visual acuity up to 2/60 (20/1000) and/or a visual field of less than 5 degrees radius. The B3 category includes individuals with visual acuity better than 2/60 (20/1000) and up to 6/60 (20/200), and/or a visual field of more than 5 degrees and less than 20 degrees radius. The B4 category, recognized by USABA and some national adapted sports programs, includes athletes with visual acuity better than 6/60 (20/200) and up to 6/24 (20/70), and/or a visual field of more than 20 degrees and up to 40 degrees radius. These classifications align with international standards for visual impairment in sport but extend to include the B4 category to represent athletes with mild yet functionally significant vision loss. All data collection occurred at a school for the blind and a recreational camp for children and youth with visual impairments in three states across the United States.

### 2.2. Materials

The VISIONS QL is a 63-item inventory designed specifically to measure the health-related quality of life for children and youth with VI [17,23]. A modified version of the original HEAR-QL [16], which was also a modification of the PedsQL [15], the VISIONS QL is a QoL measure intended specifically for those who experience vision loss. VISIONS QL includes six sub-domains (Educational Implications [9 items], Social Integration [14 items], Psycho-social Well-being [10 items], Speech, Language, Communication [10 items], Family Relationships [12 items], and General Functioning [4 items]) and requires approximately one hour to complete. Items are scored from 1 to 4 with 1 = completely disagree and 4 = completely agree and then translated into a percentage (1 = 25%, 2 = 50%, 3 = 75% and 4 = 100%). However, some questions can be reverse-coded depending upon the intention of the question (e.g., Q # 60, “Do you feel that you are independent in all activities in daily life [positive] vs. Q # 63, “Do you feel that vision loss is causing lots of restrictions in life? [negative]). All six subscales are then combined into an overall percentage of 25–100, with higher scores reflecting a higher quality of life perception. The original VISIONS QL has demonstrated evidence of content and face validity with item modification consensus agreed upon by a panel of experts [17]. However, only initial psychometrics have occurred, warranting the need for this study.

### 2.3. Procedures

The Institutional Review Board at the University of South Carolina approved all procedures (Pro00110871 and date of approval—16 August 2021, initial, current 16 August 2025). All participants were recruited via convenience sampling during the first day of the camps or through emails sent to parents at the school for the blind. Parents/guardians provided informed written consent and children/youth verbal assent. Parents then completed the demographic questionnaire with their children, including children’s self-reported gender. Next, all children/youth completed the 63-item VISIONS QL in the medium of their choice (e.g., large print, braille, 12-point font, or read aloud by a member of the research staff) during day one of camp and again at day seven of camp. All survey data and demographics were entered using a triple-pass verification process to ensure accuracy and consistency by members of the research staff. In the first pass, responses were transcribed directly from completed survey forms into an electronic database. During the second pass, the entire dataset was independently re-entered and electronically compared to the initial entry to identify discrepancies. In the third pass, all discrepancies were manually cross-checked against the original surveys, and corrections were made accordingly. This approach ensured a high degree of accuracy and minimized data entry errors prior to analysis.

#### Data Analyses

With a primary aim of variable/item reduction while maintaining as much variability as possible, we conducted a Principal Components Analysis (PCA). Thus, the primary focus was on variance extraction rather than estimating latent constructs. Although the sample size (*N* = 148) resulted in a participant-to-item ratio of approximately 2.3:1, recent simulation research indicates that stable factor solutions can be obtained with smaller samples when communalities are high and factors are well-defined [24]. Given the high KMO value (>0.80) and strong item communalities (>0.60) observed in this study, the sample was considered adequate for exploratory PCA. This approach was appropriate for our study’s exploratory purpose and the unique, limited-access population examined. Prior to conducting the PCA, we tested for outliers and missing values as well as all statistical assumptions (mentioned below). Afterwards, we then conducted a PCA with orthogonal varimax rotation to reduce dimensionality and identify components with the original 63-item VISIONS QL. Components with eigenvalues greater than 1.0 were retained and a scree plot was observed. Factor loadings above 0.40 were deemed meaningful. To explore the additional psychometric properties of the VISIONS QL Brief, we conducted three measures of reliability (McDonald’s Omega, Cronbach’s Alpha, and test–retest reliability. Internal consistency reliability was assessed using Cronbach’s alpha (α) and McDonald’s omega (ω) coefficients. Both indices estimate the degree to which items on a scale measure the same underlying construct. Values range from 0 to 1, with higher values indicating greater internal consistency. Following conventional guidelines, coefficients below 0.60 indicate poor reliability, values between 0.60 and 0.69 are questionable, between 0.70 and 0.79 are acceptable, between 0.80 and 0.89 are good, and values of 0.90 or higher are considered excellent [25]. McDonald’s ω is additionally recommended as it does not assume equal factor loadings across items and provides a more robust estimate of composite reliability compared with Cronbach’s α [26]. Test–retest reliability was evaluated using intraclass correlation coefficients (ICCs; two-way mixed effects, absolute agreement model), including subscale and total results. ICC values range from 0 to 1, with higher values indicating greater stability between administrations. Following established guidelines, ICCs below 0.50 indicate poor reliability, values between 0.50 and 0.74 indicate moderate reliability, between 0.75 and 0.89 indicate good reliability, and values of 0.90 or higher indicate excellent reliability [27,28]. All analyses occurred with SPSS v. 29. There was no missing data or outliers.

## 3. Results

Sampling adequacy was confirmed via the Kaiser–Meyer–Olkin (KMO) measures (KMO = 0.81), indicating meritorious adequacy, and Bartlett’s Test of Sphericity was significant (X^2^ = 963, df = 190, *p* < 0.001), suggesting sufficient correlations among variables for factorability. Mardia’s test of multivariate normality showed no skewness or kurtosis (*p* > 0.001). Linearity and multicollinearity were checked through inspection of the correlation matrix and variance inflation factors (VIFs), with no issues observed.

The results of the PCA yielded three components per Kaiser’s criterion (e.g., eigenvalues above 1.0) and via visual inspection of the scree plot. Factor loadings that were above 0.40 were retained (*N* = 20) and split into three components (Educational Opportunities = 7 items; Social and Familial Implications = 8 items; Communication = 5 items; see Table 1 and Appendix A). Finally, the original 63-item version showed concurrent validity with the 20-item brief scale (r = 0.92, *p* < 0.0001).

### Reliability

Overall, the resulting 20 VISIONS QL Brief had a high level of internal consistency (α = 0.857; Ω = 0.858). Additionally, there were significant associations among each subscale between time one and time two (correlations varied between ICC = 0.79–0.87; 95% CI = 0.71–0.88) and between the total scores (ICC = 0.89, 95% CI = 0.84–0.92).

## 4. Discussion

Results from this study contribute to the growing body of research on QoL among children and youth with VI by evaluating the psychometric properties of the VISIONS QL and developing a shortened version suitable for use in educational, clinical, and community settings. The findings affirm the multidimensional nature of QoL in this population and highlight the importance of reliable, context-sensitive tools for capturing the lived experiences of children with VI.

The results revealed three distinct domains: Educational Opportunities, Social and Familial Implications, and Communication. Together, these domains accounted for 46 percent of the variance. These components align with existing literature on QoL in pediatric populations, which emphasizes the interplay between academic engagement, social relationships, and self-expression [29,30]. The emergence of these domains from a sample of children and youth attending a camp or at a school for the blind, settings designed to promote independence and inclusion, suggests that the VISIONS QL Brief captures meaningful aspects of their developmental and psychosocial experiences [31].

Results from the VISIONS QL Brief demonstrated strong internal consistency and test–retest reliability, indicating that it is both stable over time and sensitive to the nuances of self-report. The high correlation between the original 63-item version and the 20-item brief scale supports the usage of the shortened measure and its potential utility in settings where time, accessibility, and cognitive load are critical considerations. These findings are consistent with prior research on the HEAR-QL, which showed that condition-specific instruments outperform generic tools like the PedsQL in detecting QoL differences among children with sensory impairments [16,32].

Given the lack of vetted QoL instruments for U.S. children/youth with VI, the VISIONS QL Brief fills an important gap. The development of VISIONS QL responds to calls for culturally relevant and ecologically valid tools that reflect the diverse contexts in which children live and learn [6]. By incorporating input from children, youth, families, and experts, and by adapting items from established measures, the VISIONS QL Brief offers a balanced approach to assessment that honors both scientific rigor and lived experience.

The implications of this work extend beyond measurement. A psychometrically vetted and concise QoL tool can inform early intervention strategies, guide individualized education plans, and support mental health referrals. VISIONS QL can also serve as a foundation for longitudinal research tracking developmental trajectories and evaluating the impact of inclusive programming. In public health and policy contexts, the VISIONS QL Brief may help quantify disparities and advocate for resources that promote equity and access for children/youth with VI.

Although this study possesses many strengths, it is not without limitations, most of which can be addressed within future research. The use of convenience sampling from sport camps and a school for the blind allowed access to a unique population of individuals with visual impairments. However, this recruitment strategy (e.g., participants were recruited from two distinct settings, a specialized school for the blind and adapted sports camps) was intentional so that we may capture a broad range of experiences with physical activity and social engagement. This strategy was designed to enhance diversity within the sample, as individuals attending sports camps often have prior experience in adapted recreation, whereas those from educational settings may have limited exposure to organized sport. Including both groups reduced bias toward any single subgroup and allowed for comparison across varying activity contexts. While these socially supportive environments may influence quality-of-life perceptions, their inclusion strengthens the representativeness of the findings by reflecting the diverse lived experiences of individuals with VI. Therefore, while findings should be interpreted with caution regarding generalizability to the broader population of individuals with VI, given the use of a convenience sample, we took as many steps as we could to minimize that risk. Furthermore, demographic data on race/ethnicity, socioeconomic status, and comorbidities were not available. Future research should aim for broader recruitment strategies that capture individuals across a wider range of backgrounds and activity levels and include such data. Future research should focus on expanding the sample to include children/youth from diverse geographic, cultural, and socioeconomic backgrounds to ensure generalizability. Additional psychometric studies, including confirmatory factor analysis, polychoric correlations (for bivariate factors) and criterion-related validity testing, will further strengthen the tool’s utility. Exploring the VISIONS QL’s responsiveness to change over time and across interventions will also be essential for establishing its role in outcome evaluation. Finally, because this study employed a cross-sectional design, causal and developmental inferences cannot be made from the observed relationships. The results describe associations among variables at a single point in time and should be interpreted accordingly. Future research using longitudinal or experimental designs is needed to explore temporal relationships and potential causal mechanisms underlying these findings. Longitudinal studies could also strengthen the test–retest reliability by reducing potential recall biases.

## 5. Conclusions

In conclusion, the VISIONS QL Brief represents a promising advancement in the assessment of QoL among children/youth with VI. Its brevity, reliability, and conceptual clarity make it a practical and meaningful tool for educators, clinicians, researchers, and policymakers committed to supporting the holistic development of children with VI.

The results underscore the need for practitioners, educators, and policymakers to prioritize accessible physical activity opportunities and supportive social contexts for children and youth with VI. These findings also highlight the importance of using vetted, population-specific measures when assessing QoL and related constructs. Future research should build on these findings through longitudinal and intervention-based designs to examine causal pathways and evaluate the long-term impact of participation in sports programs.

## Figures and Tables

**Table 1:** Rotated Structure Matrix for PCA with Varimax Rotation of a Three-Component Structure.

Items	Mean (SD)	Rotated Component Coefficients			Extraction		Reliability Coefficient (If Dropped)	
		1	2	3	Uniqueness	Communalities	Omega	Alpha
1	78.23 (26.05)	0.78			0.36	0.64	0.85	0.85
2	73.64 (27.26)	0.76			0.37	0.64	0.85	0.85
3	71.62 (28.37)	0.74			0.38	0.62	0.85	0.85
4	81.80 (24.05)	0.63			0.56	0.44	0.85	0.86
5	81.12 (25.10)	0.59			0.55	0.45	0.85	0.85
6	70.10 (28.37)	0.51			0.56	0.44	0.85	0.85
7	82.26 (22.82)	0.48			0.76	0.24	0.86	0.86
8	87.93 (22.92)		0.83		0.32	0.69	0.85	0.86
9	66.08 (28.52)		0.67		0.53	0.47	0.85	0.86
10	79.93 (25.11)		0.62		0.51	0.49	0.85	0.85
11	93.03 (17.50)		0.61		0.56	0.44	0.85	0.86
12	91.05 (19.56)		0.57		0.57	0.43	0.85	0.86
13	82.60 (23.63)		0.54		0.66	0.34	0.85	0.86
14	81.91 (23.83)		0.50		0.75	0.26	0.86	0.86
15	66.84 (28.39)		0.43		0.78	0.22	0.86	0.86
16	68.06 (27.65)			0.78	0.39	0.61	0.85	0.86
17	72.30 (25.61)			0.68	0.44	0.56	0.85	0.85
18	72.97 (25.84)			0.67	0.48	0.52	0.85	0.86
19	68.88 (26.91)			0.63	0.59	0.41	0.86	0.86
20	83.50 (19.52)			0.47	0.65	0.35	0.85	0.86
Mean	77.12 (12.99)	76.95 (17.94)	81.23 (14.77)	73.17 (17.39)			0.86	0.86
Eigenvalue		5.61	1.91	1.72				
Proportion Variance		0.17	0.16	0.133				
Cumulative		0.17	0.33	0.46				

Note: 1 = Educational Opportunities, 2 = Social and Familial Implications, 3 = Communication.

## Data Availability

The datasets generated and/or analyzed during the current study are not publicly available due to patient confidentiality and ethical restrictions. The data contain sensitive personal health information and sharing them would compromise participant privacy. Access to the data is therefore limited to the research team in accordance with institutional review board (IRB) approval and applicable data protection regulations.

## Appendix A

**Table:**

**Question**	**Completely Disagree**	**Somewhat Disagree**	**Somewhat Agree**	**Completely Agree**
**Education and Opportunities**				
1. Do you ask questions when you are unsure in class?				
2. Do you feel that you are allowed to take part in only some competitions?				
3. Does your sight stop you from learning new skills?				
4. Do you feel that because of your visual impairment you are not able to get more information?				
5. Do you feel that vision loss is stopping you from doing things that you would like to do?				
6. Do you feel that because of your vision loss, your future is limited?				
7. Do you feel that opportunities are less because of vision loss?				
**Social and Familial Integration**				
8. Do you prefer studying only with children with vision loss?				
9. Do you need somebody to be behind you to guide you always?				
10. Are your sighted parents and siblings the only sighted people you talk to?				
11. Does your family involve you in their discussions?				
12. Do you feel that your parents are worrying too much about you?				
13. Do you interact less with your family members because of your vision loss?				
14. Does your family try to hide from others that you have a vision problem?				
15. Are parents too worried about your safety?				
**Communication**				
16. Do you want some known people to tell what you need when talking to strangers?				
17. Do you have difficulty in following the conversations in a group?				
18. Do you need to ask for help to communicate anything?				
19. Do you have difficulty in concentrating for a longer conversation?				
20. Are you satisfied with the way you talk to others?				
